# The effect of sediment grain properties and porewater flow on microbial abundance and respiration in permeable sediments

**DOI:** 10.1038/s41598-020-60557-7

**Published:** 2020-02-27

**Authors:** Soeren Ahmerkamp, Hannah K Marchant, Chao Peng, David Probandt, Sten Littmann, Marcel M. M. Kuypers, Moritz Holtappels

**Affiliations:** 1 Max Planck Institute for Marine Microbiology, Celsiusstr. 1, 28359 Bremen, Germany; 2 Marum Center for Marine Environmental Sciences, Bremen, Germany; 3 University Tübingen, Center for Applied Geoscience, Geomicrobiology, Geschwister-Scholl-Platz, 72074 Tübingen, Germany; 4 Alfred Wegener Institute Helmholtz Center for Polar and Marine Research, Am Handelshafen 12, 27570 Bremerhaven, Germany; 5 Present Address: College of Life Sciences, China West Normal University, 637002 Nanchong, China

**Keywords:** Biofilms, Element cycles, Sedimentology

## Abstract

Sandy sediments cover 50–60% of the continental shelves and are highly efficient bioreactors in which organic carbon is remineralized and inorganic nitrogen is reduced to N_2_. As such they seem to play an important role, buffering the open ocean from anthropogenic nitrogen inputs and likely remineralizing the vast amounts of organic matter formed in the highly productive surface waters. To date however, little is known about the interrelation between porewater transport, grain properties and microbial colonization and the consequences for remineralization rates in sandy sediments. To constrain the effect of theses factors on remineralization in silicate sands, we incubated North Sea sediments in flow-through reactors after separating into five different grain size fractions. Bulk sediment and sediment grain properties were measured along with microbial colonization and cell abundances, oxygen consumption and denitrification rates. Volumetric oxygen consumption ranged from 14 to 77 µmol O_2_ l^−1^ h^−1^ while nitrogen-loss via denitrification was between 3.7 and 8.4 µmol N l^−1^ h^−1^. Oxygen consumption and denitrification rates were linearly correlated to the microbial cell abundances, which ranged from 2.9 to 5.4·10^8^ cells cm^−3^. We found, that cell abundance and consumption rates in sandy sediments are influenced (i) by the surface area available for microbial colonization and (ii) by the exposure of these surfaces to the solute-supplying porewater flow. While protective structures such as cracks and depressions promote microbial colonization, the oxygen demand is only met by good ventilation of these structures, which is supported by a high sphericity of the grains. Based on our results, spherical sand grains with small depressions, i.e. golf ball like structures, provide the optimal supporting mineral structure for microorganisms on continental shelves.

## Introduction

Permeable sediments cover more than 50% of the continental shelves and likely play a key role in nitrogen and carbon cycling in marine environments^1^. Advective transport occurs in permeable sediments due to the interaction of centimeter scale bedforms (such as ripples) with overlying currents. The resulting pressure gradients pump bottom water through the pore space^2,3^, providing electron acceptors and donors to highly active microbial communities, and releasing degradation products and reduced material back to the water column. Advection in permeable sands regulates exchange fluxes and accelerates bentho-pelagic coupling by several orders of magnitude in comparison to cohesive sediments^4^. However, it is only in the last two decades that rate measurements have taken advection in permeable sediments into account. Such measurements have revealed that permeable sediments in marine environments are hotspots of organic matter remineralization and denitrification^4–10^.

Within sandy sediments, microbial cell numbers range between 10^8^–10^9^ cells cm^−3^ ^11–13^. The vast majority of these cells (>99%) are attached to sand grains^14–16^, primarily in concave regions that are protected from abrasion during sediment transport and resuspension^17^. The microbial community is exposed to different electron acceptors and donors in these microenvironments dependent on the advective transport regime. For example, changes in bottom current velocity directly affect the velocity of pore water advection, which impacts oxygen penetration depths and redox conditions within the sediment^4^. These dynamic redox regimes promote facultative anaerobes and overlaps in metabolic processes such as aerobic respiration and denitrification^7,18^. While advective transport is the primary factor determining the concentrations of solutes in the vicinity of the attached microbial community, transport into the microenvironments on the sand grains and ultimately to the microbial cells is governed by diffusion. This may lead to solute concentrations near the microbes that differ from the bulk porewater, possibly causing diffusion limitation^19,20^. Quantification of remineralization rates, therefore, requires experimental designs that mimic the natural porewater advection.

In the last decade, our understanding of sandy, permeable sediments has been advanced by the use of flow-through reactors (FTRs) that allow microbial reactions to be studied under advective conditions^18,21–24^. Volumetric oxygen respiration rates in sands have been found to range in between 10–250 µmol l^−1^ h^−1^ and nitrogen-loss via denitrification ranges between 1–15 µmol l^−1^ h^−1^ ^5,21,23,24^. Oxygen consumption rates in permeable sands appear to be stimulated by increases in porewater advection and by addition of dissolved organic matter to the inflowing water^25,26^. So far, little is known about how sand grain microenvironments that microorganisms inhabit affect respiration rates. On one hand, life in cracks and depressions on the surface of sand grains protects microorganisms from abrasion. On the other hand, it is more likely that bacterial consumption rates at these locations are limited by diffusive transport. In order to understand how cell abundances and respiration rates are affected by this diffusive transport, the sphericity of sand grains, i.e. their deviation from a spherical surface due to cracks and depressions, must be considered. In addition to the local conditions on the individual sand grain, the grain size distribution itself could play a role in microbial colonization, as retention of particulate organic matter is more likely when small sand grains narrow the pore space. Such patchy distribution of organic matter likely shapes the distribution of microbial cells attached to the sand grains. Thus far, most studies on microbial distribution patterns in sands do not include FTR rate measurements and vice versa.

This study investigates how sand properties, such as grain size, surface area and sphericity, influence microbial distribution and microbial respiration rates under advective conditions. We collected sandy sediments from the subtidal North Sea and separated the sediment into 5 different grain size fractions. The different fractions were exposed to a range of porewater velocities in FTRs while measuring rates of oxygen consumption, denitrification and NOx (nitrate and nitrite) production. In addition, we determined cell abundances and organic carbon content as well as sediment properties such as dispersion, permeability, porosity, grain size, surface area and sphericity, and correlated these to cell abundance and respiration rates.

## Methods

### Study site

Sampling was conducted in the subtidal North Sea during RV Heincke cruise 432 in autumn 2014. The sampling site (GPS 54 °10.48′N, 7° 57.44′E) was located in the German Bight between Helgoland and Bremerhaven, where nutrient supply of the rivers Weser and Elbe is fueling enhanced primary production^27^. The water depth was around 20 meters and typical bottom water velocities range between 7–21 cm s^−1^ with an additional contribution of the wave orbits by up to 8 cm s^−1^ ^4^. The water-temperature during the sampling campaign was 17 °C. Sediments were collected using repeated Van Veen grabs. The upper two centimeters of the sediment were sampled and placed into transport boxes. The procedure was repeated approx. 30 times until sufficient amounts of sediment were sampled. The samples were completely immersed in sea water and additional bottom water was collected and stored in the dark. Organic carbon contents in the water column were on average 0.1 mg POC l^−1^ (pers. comm. Andreas Neumann, HZG).

### Experimental setup

In the lab and approximately 24 hours after retrieval, the sediment was gently homogenized and separated into different grain size fractions using sieves with mesh sizes of 1000 µm, 710 µm, 500 µm and 355 µm in sequential order. This resulted in four grain size fractions that varied in between 355 µm – 500 µm, 500 µm − 710 µm, 710 µm – 1000 µm and <355 µm which approximately reflects the phi scale for sediments (see also Fig. SI 1). The grain size distributions of the fractions were roughly delimited by the mesh size but tailed towards larger grain sizes, which is further discussed below. Four sorted sediment fractions and the original (mixed) sediment were each filled into a flow through reactor (FTR) that was completely submersed in sea water to avoid trapping of air bubbles. The flow through reactors have the advantage that the advective porewater flow can be controlled and microbial processes can be estimated to high accuracy^3,21,22,25,28^. The FTRs has an inner diameter of *d* = 10 cm and a length of *L*_*C*_ = 20 cm. This relatively large volume minimizes bias caused by pore water flow along the walls and ensures that enough sediment is present in the FTR to capture the heterogeneity. The length of the flow paths is similar to the typical streamlines length in the upper few centimeter of the permeable sediment^29,30^. Radial grooves at the inlet lid and outlet lid of the reactor allowed for a homogenous percolation. Sediment discharge into the tubing was avoided by covering the inlet and outlet lid with a plankton net (80 µm mesh).

The 5 FTRs were positioned vertically, and were continuously supplied with air saturated seawater from 20 L Duran bottles using polyetheretherketone tubing (PEEK) (approx. 3 cm) and TygonTM tubing (approx. 20 cm). The core percolation was driven by peristaltic pumps (ISMATEC, Reglo digital MS-4/6) adjusted to reproduce porewater velocities between 1 cm h^−1^ to 30 cm h^−1^ as typically found in permeable sediments (Huettel 1996)^25^. The cores and reservoirs were kept in the dark at a temperature of 19 °C. Oxygen respiration and NOx (nitrate and nitrite) production rates within the 5 cores were measured at 11 different flow rates over the next 21 days.

### Flow characteristics

To assess the homogeneity of the flow within the FTRs and to estimate flow dispersion and sediment porosity, argon saturated seawater was supplied and an argon-breakthrough curve was measured using a membrane-inlet mass spectrometer. The argon-breakthrough represents the cumulative of the porewater age distribution, of which the first moment is the mean retention time *r*_*t*_ and the second moment the longitudinal dispersion *D*_*L*_ = *α*_*L*_*u*, with *α*_*L*_ the longitudinal dispersion coefficient. To allow for a better comparison, the time variable *t* and longitudinal dispersion *D*_*L*_ were non-dimensionalized using the mean retention time, core length *L*_*c*_ and porewater velocity *u*: t* = *tr*_*t*_^−1^*, α*_L_* = *D*_*L*_*u*^−1^*L*_*c*_^−1^ = *α*_*L*_*L*_*c*_^−1^, where an asterisk denotes non-dimensional variables. For the 1-D scalar-transport equation and boundary conditions as present in the flow-through reactors an analytical solution exists^28^:

1 $$\frac{C}{{C}_{0}}=0.5\cdot {\rm{erf}}\,(\frac{1-{t}^{\ast }}{2\sqrt{{\alpha }_{L}^{\ast }{t}^{\ast }}})$$

This function is similar to the cumulative function of the Gaussian distribution, where $\sigma =\sqrt{2{\alpha }_{L}^{\ast }}$ is the standard deviation, C the measured argon concentration at the outlet and C_0_ the inlet concentration of argon. Thus, most studies have used Eq. 1 as a fit function for the breakthrough curves^31^. Here, we directly estimate the unknown sediment porosity via the retention time and the flow dispersion from the second moment of the porewater age distribution^28^.

When measuring reaction rates in FTRs the reactants should not be depleted before the porewater reaches the outlet. We employ the Damköhler number to express the specific rates of reaction and transport (see also appendix SI5):

2 $$Da=\frac{R}{{C}_{in}}\cdot \frac{L\theta }{u}$$

where L is the length scale, θ the porosity, R the volumetric oxygen consumption and C_in_ the reservoir oxygen concentration. In case Da >1 the solute would be depleted within the core. To account for flow dispersion the longitudinal dispersion coefficient is added to the core length $L={L}_{c}+\sqrt{2{\alpha }_{L}u{r}_{t}}$ causing *L* to be 10% to 40% larger than the core length (*L*_*c*_) itself. This is the length scale to which dispersion would affect the respiration rate measurements.

### Sediment characterization

Grain size distributions from the size fractionated sediments were measured using a laser diffraction particle size analyzer (Beckman Coulter, LS 200) and binned into 92 size classes ranging from d_0_ = 0.4 µm to d_1_ = 2000 µm. Prior to measurements the samples were acidified to remove carbonates. From the measured grain size distribution the theoretical surface area to volume ratio *S*_*V,T*_ can be calculated:

3 $${S}_{V,T}=6(1-\theta ){\int }_{{d}_{0}}^{{d}_{1}}\frac{{\rm{p}}({d}_{g})}{{d}_{g}}d{d}_{g}$$

where *θ* is the porosity, p(*d*_*g*_) is the probability density function of the respective sediment fractions and *d*_*g*_ the diameter of the grains. Equation 3 assumes spherical grains, which is not necessarily the case. Therefore, the surface area per mass (*Am*^−1^) was also measured via nitrogen adsorption using a Brunauer-Emmett-Teller analyzer (Quantachrome Quantasorb Jr.) (see also Mayer^12^). Based on density *ρ*_*p*_ of the particles the surface-to-volume ratio can be calculated:

4 $${S}_{V}={\rho }_{p}\frac{A}{m}(1-\theta )$$

The permeability of the sediment fractions was estimated by measuring the pressure drop along the flow through reactor. Along the flow direction two tubes were attached to the FTR in *δ* = 10 cm distance. The pressure drop is visible as a difference between the water heads (*h*_1_*-h*_0_) in the tubes. Based on Darcy’s law the porewater velocity can be calculated:

5 $$u=\frac{k}{\mu {\rm{\theta }}}\nabla p$$

where *k* is the permeability, *μ* the dynamic viscosity and ∇*p* the pressure gradient. The pressure gradient can be determined by the hydrostatic pressure drop between the tubes: $\nabla p=\rho g({h}_{1}-{h}_{0}){\delta }^{-1}$, where *ρ* is the water density, *g* the acceleration by gravity. Substitution and rearranging yields:

6 $$k=\frac{u\mu \delta }{\rho g({h}_{1}-{h}_{0})}{\rm{\theta }}$$

The sphericity of sand grain is defined as the ratio of the surface area of a sphere to the surface area of the sand grain at the same volume^32^. However, accurate determination would require 3D-reconstructions of individual sand grains which would severely limit the feasible sample size. To allow for larger throughput of sand grains, we estimated the sphericity in a 2D plane by determining the perimeter of the sand grains relative to the perimeter of a circle at the same area. Sand grains were subsampled from the flow through reactors and transferred onto petri dishes for determination of 2D-sphericity. Photographic images were taken at 25x magnification using a SLR camera (Nikon D90) attached to an inverse microscope (Leica DMI 6000 B).

The images were processed using the image processing toolbox of Matlab (Mathworks 2018 b). Briefly, the image was inverted and binarized using a threshold of 0.8. In the resulting images, sand grains appeared as connected white areas, which were identified using an automated script, and subsequently the edges of the connected white areas were traced (see Fig. SI 4b). These edges represent the perimeter p of the sand grains. The area A was determined by integrating the connected white pixels. Based on A and p the sphericity indicator *S*_*P*_ can be determined ${{\boldsymbol{S}}}_{{\boldsymbol{P}}}=\frac{\sqrt{2{\boldsymbol{\pi }}{\boldsymbol{A}}}}{{\boldsymbol{p}}}$. In total 45 images were taken and 5–15 sand grains were processed per image. In total, approximately 400 sand grains were individually analyzed.

For carbon content analysis, sediment samples were taken from the FTRs and frozen at −20 °C. The sediment samples were dried, ground, acidified, and subsequently the total organic carbon content was determined using an elemental analyzer (Elementar, vario TOC cube).

### Cell counts

After ten days of incubation, the FTRs were opened for a short time period and 5 cm of sediments were removed, then 5 ml sediment samples were taken to quantify the total cell abundance. The FTRs were subsequently closed again, taking care that no preferential pathways had been created. Approximately 0.5–1 ml of sediment were fixed in a formaldehyde solution (1% in phosphate buffered saline, PBS) at room temperature followed by repeated centrifugation and replacement of the supernatant with the PBS solution. Cells were then extracted from the grains by sonication (Sonopuls HD70) with a UW 70 sonication probe (Bandelin) following the protocols of Probandt *et al*.^33^ and Kuwae, T. and Hosokawa, Y.^34^. Each sample was sonicated 5 times for 30 seconds at a low intensity (25% power, cycle 20). Supernatant was collected and replaced by the PBS solution between the sonication steps. Subsequently, 200 µl to 500 µl of supernatant was subsampled and diluted in 10 mL PBS:EtOH solution. Cells were then filtered on 0.2 µm Isopore membrane filters (Millipore). Finally, filters were dried at 40 °C and embedded in 0.1% agarose gel (Biozym, low melting temperature gel with strength larger than 800 g cm^−2^) and stored at −20 °C.

Cells were enumerated after staining with SYBR Green I (invitrogen). The 10000x SYBR Green I stock solution was diluted 1:50 in sterile 1 mol l^−1^ Tris HCl with a pH of 7.5. Subsequently, 60 µl were transferred onto a petri dish. Filter pieces were stained for 20 minutes and stabilized in mounting medium (Mowiol 4–99 Sigma). Cells were counted using an epifluorescence microscope (Leica DMI6000B) at an excitation wavelength of 498 nm. For each filter 28 to 61 randomly chosen grids were counted, encompassing an area of 1000 µm^2^ with at least 1300 SYBR Green signals in two biological and three technical replicates.

### *In situ* colonization on sand grains

Scanning electron microscopy was used to visualize the microbial community on surfaces of sediment grains. Sediment was fixed in 2.5% glutaraldehyde in 0.1 M sodium cacodylate buffer at pH 7.2 and post fixed in OsO4 in the same buffer. Sediment was then washed in ethanol-water mixtures with increasing concentration (30%, 50%, 70%, 90% and 100%) for 15 minutes at each step, and then dried in a critical point dryer (Leica EM CPD 300). The sand grains were then sprinkled onto double sided conductive carbon adhesive tabs. Imaging was performed using a Quanta 250 FEG scanning electron microscope (FEI) with an acceleration voltage of 2 kV. The grey scale scanning electron micrographs were colorized using Photoshop (Adobe, CS 9) to identify bacteria and detritus.

### Oxygen respiration, denitrification and combined nitrate- nitrite production

Oxygen respiration, denitrification and combined nitrate- nitrite production were estimated by measuring the difference of the respective solutes between inlet (C_in_) and outlet (C_out_) of the flow through reactor:

7 $$R=\frac{({C}_{out}-{C}_{in})}{{r}_{t}}$$

where ${r}_{t}={L}_{c}{u}^{-1}$ is the mean retention time, with *L*_*c*_ the length of the core, *u* the porewater velocity, calculated from the pump volume f $(u=f{A}^{-1}{\theta }^{-1})$, and θ the porosity.

Oxygen was continuously measured using optode flow-through cells (OXFTC, Pyroscience coupled to a 4-channel Firesting amplifier, Pyroscience). For each of 11 different flow rates, oxygen consumption was measured simultaneously on the five different sediment fractions. Flow rates were typically changed after 24 hours. After adjusting the pump volume stable consumption rates indicated that a steady state had been reached. This occurred typically after 1.5 times the mean retention time, i.e., the time a water parcel travels from inlet to outlet. Various flow rates were applied in a random order to ensure that changes in oxygen consumption rates were related to change in flow rates, rather than to temporal changes of the microbial community or organic carbon content. Furthermore, in a number of cases, rate determinations were repeated based on randomly chosen flow rates.

Nitrification, anammox, DNRA and denitrification have all been shown to occur in sandy sediments, however in the subtidal sands of the North Sea, nitrification and denitrification are the dominant processes^5^. Therefore, we focus on nitrification under oxic conditions and denitrification rates under anoxic conditions. During oxic incubations, inlet and outlet porewater was subsampled for NOx concentrations and frozen at −20 °C. Nitrite and nitrate concentrations were measured on a CLD 60 Chemiluminescence NO analyzer (Ecophysics)^35^ and rates were calculated according to Eq. 7.

After 3 weeks of oxic incubation, the seawater reservoir water was degassed by purging with argon and amended with 70 µmol l^−1 15^NO_3_^−^ and subsequently Ar, ^28^N2, ^29^N2 and ^30^N2 was continuously measured at the outlet via a membrane-inlet mass spectrometer (GAM200, IPI). Denitrification rates were calculated based on the equations described in^36^. For the denitrification experiments only one flow rate was used resulting in porewater velocities between 9 cm h^−1^ to 12 cm h^−1^ depending on the porosity of the respective sediment fractions. These porewater velocities represent an intermediate range of measured porewater velocities in nature^25^.

## Results

### Sediment properties

The median grain size of the sediment fractions ranged from 277 µm to 882 µm after sieving; similar to the mesh sizes of the sieves (Fig. SI 1, 2, Table 1). The grain size distributions indicate slight variations in sediment sorting. The original, unsieved sediment fraction (hereafter referred to as the mixed fraction) had the widest distribution, i.e. largest standard deviation, followed by the largest grain size fraction. As the grain size decreased, the distribution became narrower. Porosity was neither directly correlated to median grain size nor to sorting (Table 1). However, wide grain size distributions seem to decrease the porosity due to compactation^37^. In general, the distributions tailed towards larger grain sizes, which likely results from inaccuracies of the laser diffraction analysis and heterogeneities of sand grain aspect ratios, that allow larger grains to pass smaller mesh sizes. Together these results indicate a heterogeneous sand grain morphology, which was analyzed using imaging techniques to determine grain sphericity.

**Table 1:** The sediment characteristics for the different sediment fractions are depicted.

Fraction	Grain Size	Sorting	Sphericity	Permeability	Porosity	Longitudinal Dispersion	S_v_ (m² cm^−3^)	
	Median (µm)			(m²)		(m)	Sphere equivalent	Measured
1 (mixed)	496	2.2	0.57 (0.09)	6.6(1.1)·10^−12^	0.30 (0.02)	3·10^−3^	1.3·10^−2^	0.51(0.05)
2 (<355 µm)	277	1.4	0.60 (0.07)	6.5(0.6)·10^−12^	0.35 (0.02)	4·10^−3^	7.8·10^−3^	0.44(0.04)
3 (355 µm – 500 µm)	481	1.4	0.53 (0.05)	4.2(0.3)·10^−11^	0.41 (0.02)	7·10^−3^	5.3·10^−3^	0.13(0.01)
4 (500 µm − 710 µm)	621	1.5	0.48 (0.05)	1.9(1.4)·10^−10^	0.45 (0.02)	5·10^−3^	3.9·10^−3^	0.13(0.01)
5 (710 µm – 1000 µm)	882	1.6	0.56 (0.05)	1.5(0.8)·10^−10^	0.42 (0.02)	8·10^−3^	6.4·10^−3^	0.32(0.03)

The median grain size was measured from laser diffraction. Sorting represents the uniformity coefficient of the sediment distribution. The permeability was determined from the pressure drop along the flow through reactor for three different porewater velocities (Eq. 6) with the standard deviation in brackets. Porosity and longitudinal dispersion were extracted from the fitted breakthrough curve (see text). The surface-to-volume ratio was measured using the Brunnauer-Emmett-Teller method (accurate to 10%) and additionally calculated using Eq. 3 assuming spherical grains.

The average grain sphericity for the different sediment fractions ranged between 0.45 and 0.62 (Fig. SI 4a) and was significantly correlated to the porosity (θ = −0.62 S_P_ + 0.79, R^2^ = 0.62, t-test: p < 0.05). The negative correlation suggests that pore space volume is not only regulated by sediment sorting and median grain size, but also by the sphericity of the grains.

Permeability in the different fractions covered two orders of magnitude ranging between 6.5·10^−12^ m^2^ to 1.9·10^−10^ m^2^ (Table 1), and are within the typical range of permeable sediments as defined by Huettel *et al*. 2014. For the sieved sediment fractions the permeability increased with increasing median grain sizes. The mixed sediment had a comparably low permeability which might be caused by the denser packing resulting from the wide sediment distribution^38^. Repeated experiments with varying porewater velocities gave similar permeability results, indicating that increasing porewater velocities did not affect permeability measurements.

For a more precise description of the homogeneity of porewater flow, breakthrough curves were determined following Rao *et al*. 2007 (Fig. SI 2b). The breakthrough curves of the various sediment fractions indicated that there was no buildup of preferential pathways within the FTRs, nor were there dead spaces, i.e, volumes that were not percolated. This dead volume would be visible as a prolonged tail during the breakthrough. The longitudinal dispersion coefficients ranged between 3·10^−3^ m to 8·10^−3^ m, which are of typical pore space magnitudes. Thus, smearing of solute distribution within the core was mainly induced by shear-dispersion along the boundary layer of the grains^37^. This is also reflected by the high correspondence to the analytical breakthrough model. The breakthrough curves also reflect the distribution of flow path lengths inside the flow through reactor. During the experiments the path length variation was up to 25% of the core length (based on the second moment of the porewater age distribution, Fig. SI 2b). Estimating the flow path length is essential for the rate estimations as it allowed us to ensure that oxygen and nitrate were not consumed to less than 25% of the inflow concentrations, otherwise the reaction rates would be biased by the depletion of the electron acceptors along some of the flow paths.

Using the Brunauer - Emmett - Teller method based on dinitrogen sorption, the surface area to volume ratio was found to range in between 0.13 m^2^ cm^−3^ − 0.51 m^2^ cm^−3^ (Table 1). The largest surface-to-volume ratio was found for the mixed sediment fraction (0.51 m^2^ cm^−3^) followed by smallest sediment fraction (0.44 m^2^ cm^−3^) and the largest sediment fraction (0.32 m^2^ cm^−3^). The midrange fractions had the smallest surface-to-volume ratios around 0.13 m^2^ cm^−3^. The trends between the sediment fractions seem to be mainly induced by the combined effect of compaction, i.e. porosity, and the grain diameters. This is confirmed by the theoretical surface-to-volume ratio, which predicts similar trends at values between 0.0039 m^2^ cm^−3^ – 0.013 m^2^ cm^−3^. Despite the similar trends the calculated values deviated by factors between 25 and 56. This indicates that the surface-to-volume ratio was not solely regulated by the diameters of the grains and that there are additional factors increasing the surface-to volume-ratio, such as cracks and depressions, i.e. grain roughness^39^, and additional surface area due to iron or manganese oxide coatings.

### Sediment grain colonization and cell counts

**Figure 1:**
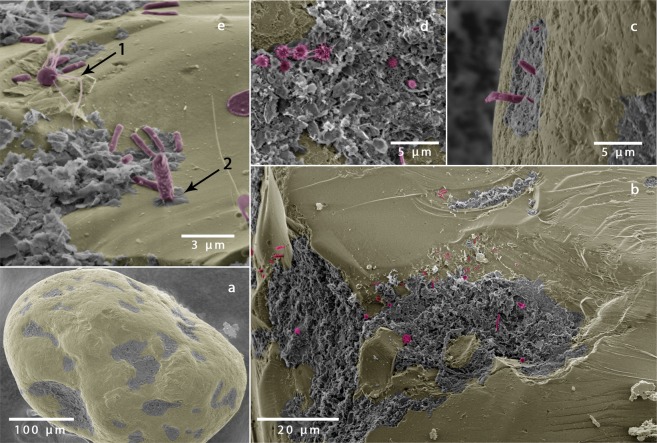
Scanning electron micrographs of the microbial colonization of a single sand grain. (**a**) Shows a quartz sand grain with undefined material (grey) in cracks and depressions and the mineral surface in yellow. (**b**–**e**) Magnifications of the cracks and depressions reveal a dense microbial colonization (magenta) with different attachment methods: e.1 shows a round shaped bacterium with pili, e.2 others attach themselves vertically or produce nets of polymers (see also^47^).

The colonization of sediment grains was investigated using secondary electron microscopy. Microbial colonization of sediment grain surfaces was visible on most sand grains. Cells of coccoid, rod-like and filamentous morphology were found with maximum dimensions of 1.2 µm, 2 µm and 25 µm, respectively. Microbial cells covered the sand grains unevenly, being particularly dense in cracks and depressions of sediment grains (Fig. 1). Grains with few fine structures and irregularities were less colonized. Since handling of sediment grains was done with greatest caution, the results indicate that microbial cells colonize sheltered regions on the sediment grain. Magnification of these sheltered regions revealed a denser microbial colonization close to undefined organic and inorganic material using a variety of attachment methods, such as pili or nets of polymers (Fig. 1d).

**Figure 2:**
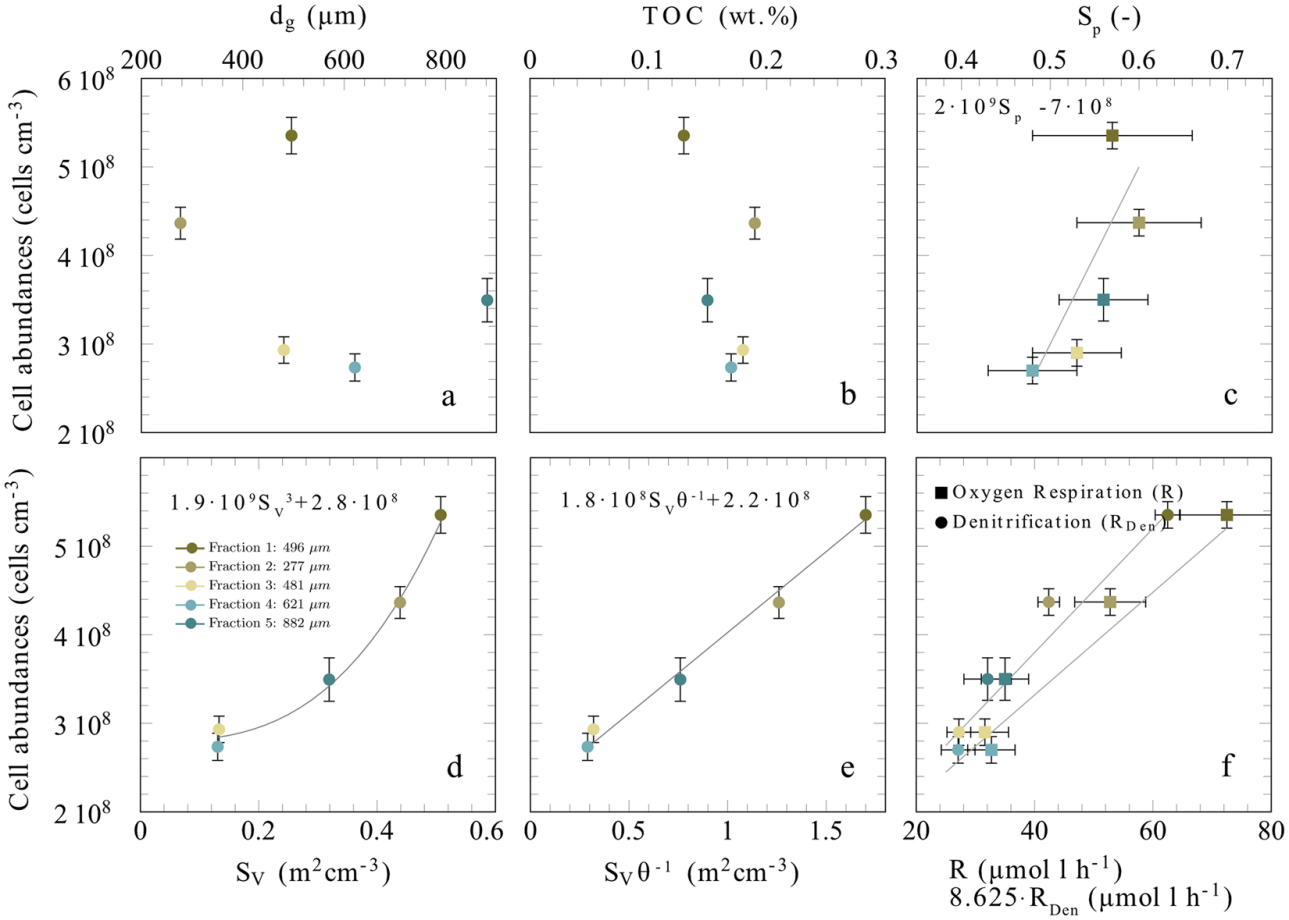
(**a**,**b**) cell abundances are not significantly correlated with median grain size nor with total organic carbon content (R^2^ < 0.2). (**c**) Sphericity (S_p_) and cell abundance show a positive correlation (R^2^ = 0.65) indicating that a more spherical grain shape promotes colonization. (**d**) The cell abundances correlate to the measured surface-to-volume ratio following a powerlaw (R^2^ = 0.93). (**e**) The correlation is linear when normalizing the surface-to-volume ratio by the porosity (R^2^ = 0.99). (**f**) Cell abundances are directly correlated to the volumetric rates of oxygen respiration (R = 7⋅10^−7^ cells − 10.5, R^2^ = 0.95) and denitrification (R_Den_ = 2⋅10^−8^ − 1.5, R^2^ = 0.95), based on a porewater velocity of 8-10 cm h^−1^. Notice, the denitrification rates are scaled by a factor of 8.625, i.e. Redfield ratio 138:16, see text for more information. In f, the error bar denotes the residual deviation from the linear trend shown in Fig. 4 for oxygen and range of denitrification measurements.

Cell numbers were determined for the five different sediment fractions and varied in between 2.9(0.1)·10^8^ cells cm^−3^ for the fraction with 481 mean diameter and 5.4(0.2) 10^8^ cells cm^−3^ for the mixed fraction (Table 2). Mean cell abundances differed significantly from each other (ANOVA, p<0.01, based on technical replicates of the cell counts) except for the midrange fractions with median grain sizes of 481 µm and 621 µm (ANOVA, p = 0.3). No trend between cell abundances and sieving iterations were observed, indicating that mechanical abrasion induced by the sediment processing did not affect cell abundances. Cell abundances were correlated neither to mean grain diameter nor to organic carbon content (Fig. 2a,b). However, a strong positive correlation between sphericity and cell counts was observed (Fig. 2c). In addition, the cell numbers were strongly correlated to the surface-to-volume ratio (Fig. 2d). Based on this relationship the average areal cell density was estimated to range in between 6.4·10^−3^ cells µm^−2^ to 1.4·10^−2^ cells µm^−2^. This is equivalent to a characteristic area of 156 µm^2^ to 71 µm^2^ per cell. Qualitatively, these cell numbers are confirmed by the scanning electron microscopy.

**Table 2:** Microbial reaction rates per volume of porewater for the different grain size fractions.

Fraction	Total Organic Carbon	Median Grain Size	Cell Counts	Oxygen Respiration		Denitrification	NO_X_ Production
	wt.%	(µm)	(cells cm^−3^)	(µmol l^−1^ h^−1^)		(µmol l^−1^ h^−1^)	(µmol l^−1^ h^−1^)
1 (mixed)	0.13	496	5.4(0.2)·10^8^	62(2)	65* (3)	8.4(0.8)	5.8(0.8)
2 (<355 µm)	0.19	277	4.4(0.2)·10^8^	42(2)	43* (2)	6.1(0.2)	4.4(0.8)
3 (355 µm – 500 µm)	0.18	481	2.9(0.1)·10^8^	27(2)	26* (1)	3.7(0.3)	3.3(0.5)
4 (500 µm − 710 µm)	0.17	621	2.7(0.2)·10^8^	27(3)	27* (2)	3.8(0.1)	2.3(0.5)
5 (710 µm – 1000 µm)	0.15	882	3.5(0.3)·10^8^	32(4)	32* (3)	4.1(0.4)	2.4(0.4)

The cell numbers are presented in cell counts per cubic centimeter of wet sediment. The oxygen respiration rates are shown as averages and those marked by (*) were measured at the porewater velocity of the denitrification experiments. The numbers in brackets denote the range for denitrification experiments (2 technical replicates), standard deviation of residuals for oxygen respiration, standard error for cell counts and standard deviation for nitrate- nitrate production.

When cell counts from all sediment fractions were compared, cell abundance increased with both calculated and measured surface-to-volume ratio, following a power-law function with an exponent of 3 (Fig. 2d). When the surface-to-volume ratio was divided by porosity, the correlation with cell abundance became linear (Fig. 2e).

### Oxygen respiration

**Figure 3:**
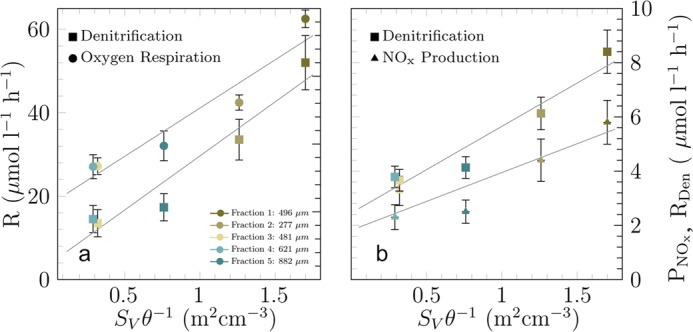
(**a**) The oxygen respiration rates and denitrification rates are shown against the surface-to-volume ratios normalized by the porosity. The oxygen respiration rates presented are based on a porewater velocity of 8–10 cm h^−1^. The error bar denotes the residual deviation from the linear trend shown in Fig. 4 for oxygen and range of denitrification measurements. (**b**) Also the NOx production rates follow a similar slope and are only slightly smaller compared to the denitrification rates. The trends of the production and consumption rates are well represented by linear fit (*R* = 23.2 *S*_*V*_ θ^−1^ + 18.0, R^2^ = 0.92 for oxygen respiration, for denitrification *R*_*Den*_ = 3.2 *S*_*V*_ θ^−1^ + 2.5, R^2^ = 0.92 and *P*_*NOx*_ = 2.3 *S*_*V*_ θ^−1^ + 1.8, R^2^ = 0.80 for NO_X_ production).

Seawater was pumped through the FTRs at porewater velocities of 1 cm h^−1^ to 30 cm h^−1^, which are representative of those measured in continental shelf sediments^25^. The bulk oxygen respiration rates varied by a factor 5.5, ranging between 14 µmol l^−1^ h^−1^ and 77 µmol l^−1^ h^−1^, and were significantly correlated to the cell abundances (Fig. 2e) and surface-to-volume ratios of the sand fractions in the flow-through reactors (Table 2, Fig. 3a,b). Similar to cell abundance, the correlation was non-linear but became linear when surface-to-volume ratio was normalized by porosity.

**Figure 4:**
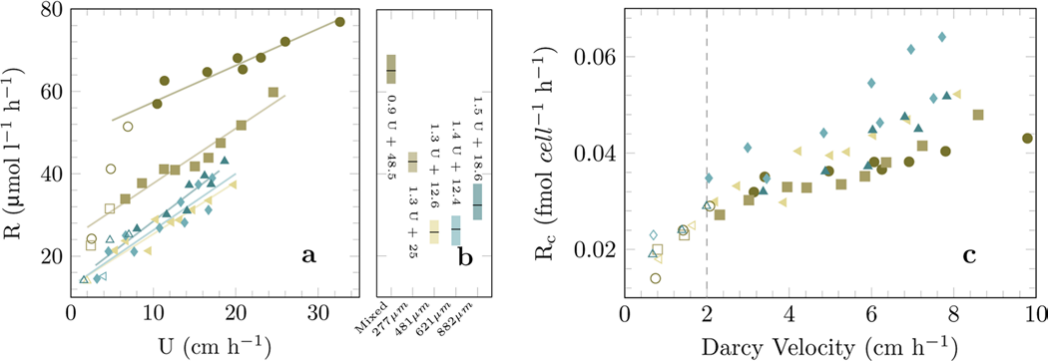
(**a**) The increasing respiration rates (R) are shown against the porewater velocity (U). Measurements where oxygen was completely consumed within the core, or which are affected by dispersion (i.e. Da >1), are shown as open symbols. For the remaining measurements the respiration rates increase linearly with the imposed porewater velocity. (**b**) The equations for the linear regression are shown. (**c**) By normalizing the respiration to single cells (assuming oxygen respiration by every cell) respiration rates coincide (R^2^ > 0.90 for all equations). The porewater velocity is scaled by the porosity to the Darcy velocity which leads to a coinciding critical Darcy velocity around 2 cm h^−1^.

When normalizing bulk respiration rates with cell numbers, the cell specific respiration was similar in all grain size fractions, varying between 0.03 fmol cell^−1^ h^−1^ to 0.06 fmol cell^−1^ h^−1^, mostly due to porewater velocity (Fig. 4c). This calculation assumes that all bacterial cells respire oxygen, which was probably not the case, but it allows for normalization by assuming that the relative amount of oxygen respiring cells stays similar.

In all sediment fractions, oxygen respiration rates increased with porewater velocities (Fig. 4a). This velocity effect was not related to the order in which the experiments were run, ruling out an adaptation of the microbial community over time. The velocity effect, here defined as the slope of increase in Fig. 4a, was different between the sediment fractions ranging from 0.9 to 1.4 µmol l^−1^ cm^−1^. There was no clear relationship between the velocity effect and the mean grain size. However, the velocity effect was positively correlated with porosity (Fig. SI 4).

Total organic carbon contents (TOC) of the sediment fractions varied little with values ranging between 0.13 and 0.19 wt.%. TOC contents increased significantly with the better sorted (i.e. smaller sorting coefficient: *s*_*sed*_) sediment fractions (*o*_*c*_ = −0.066·*s*_*sed*_ + 0.27, R^2^ = 0.83). Nevertheless, there was no significant correlation with oxygen respiration, denitrification or NO_x_ production (t-test: p < 0.05).

### NO_x_ production

The remineralization of organic matter results in a release of ammonium, which can then be oxidized via nitrite to nitrate by nitrifying microorganisms under oxic conditions. Assuming a biomass composition that follows the Redfield ratio and that all ammonium is oxidized to NO_x_, the NO_x_ production based on the oxic remineralization would follow a ratio of 16:138 for N:O_2_^40,41^. Reservoir NO_x_ concentrations averaged 7 µmol l^−1^ h^−1^. The average NO_x_ production rates in the flow through reactors ranged between 2.3 µmol l^−1^ h^−1^ and 5.8 µmol l^−1^ h^−1^ (Table 2). The rates of oxygen respiration and NO_x_ production were significantly correlated at a slope of 0.102 (R^2^ = 0.4, t-test: p = 6·10^−8^), which is only slightly below the expected Redfield-Ratio of N:O_2_ = 16:138 = 0.1159. Similar to oxygen respiration rates, the NOx production rates correlated non-linearly with grain surface-to-volume ratios and linearly when porosity was considered (Fig. 3a,b).

### Denitrification

In order to determine denitrification rates, sea water was degassed and amended with ^15^N-labelled nitrate. Experiments were performed at constant pumping speed which translates into porewater velocities of 9 cm h^−1^ – 12 cm h^−1^, depending on the different sediment properties. The denitrification rates were roughly an order of magnitude below the oxygen respiration and ranged in between 3.6 µmol l^−1^ h^−1^ to 8.4 µmol l^−1^ h^−1^. The rates of oxygen respiration and denitrification were significantly correlated at a slope of 0.14 (R^2^ = 0.98), which is only slightly above the expected value of Redfield N:O_2_ = 16:138 = 0.1159. The denitrification rates under anoxic conditions were similar to the NO_x_ production rates under oxic conditions (Fig. 3), implying that a large fraction of the produced nitrate would be reduced under anoxic conditions. Similar to oxygen respiration rates, the denitrification rates correlated non-linearly with grain surface-to-volume ratios and linearly when porosity was considered (Fig. 3a,b).

## Discussion

Volumetric oxygen respiration rates (up to 77 µmol l^−1^ h^−1^) and denitrification rates (up to 8.4 µmol l^−1^ h^−1^) were within the range of rates measured recently in continental shelf sediments^4,5,8,21,23^. The oxic NOx production (i.e. the nitrification of ammonium) was correlated to oxygen consumption following a slope close to the Redfield ratio of O_2_:N = 138:16. Similar to previous observations (e.g.^23^) the measured denitrification rates were significantly correlated to oxygen respiration following a similar trend with a slope of 138:16 (Fig. SI 3). We conclude that denitrification was closely coupled to the NOx production. Most of the ammonium released during remineralization, therefore, has the potential to be nitrified, subsequently denitrified and removed from the fixed nitrogen pool^5^.

Quantification of cell numbers within sandy sediments are rare. Most have thus far relied on bulk quantification of cell numbers, neglecting the variation in structure and size between individual grains. Here, we determined cell numbers to be around (2.7–5.4·10^8^ cells cm^−3^), which is in the range of the few counts reported previously (1·10^8^ cells cm^−3^ to 2.3·10^9^ cells cm^−3^ ^11,14,26^. The cell counts did not correlate directly with the median grain size in each fraction; however, they were strongly correlated to the measured available surface area per sediment volume. Therefore the areal cell densities (10^−2^ − 10^−3^ cells µm^−2^, on average 3·10^−3^ cells µm^−2^) were similar between all sediment fractions, suggesting that the sand grains examined in this study were colonized to the same extent, regardless of their size. The absolute number of cells per sand grain (9000 to 180000 cells per grain for median grain sizes 277 µm and 885 µm, respectively) is similar to those estimated previously for single sand grains using SYBR green staining^33^. Surprisingly, the cell densities on the medium to coarse sands in this study were very consistent with those measured across a large spectrum of grain sizes, ranging from silts to fine grained sands^42^ and pebbles^43,44^, all of which have a cell density around 5·10^−3^ cells µm^−2^. This constant colonization of sediments may facilitate the incorporation of benthic remineralization rates in modelling studies focusing on coastal marine^30^ and fluvial systems^9,10,45^.

The cells were not evenly distributed on the grains, with large patches of the grains only sparsely colonized (Fig. 1). Most of the bacterial cells were found within cracks and local depressions on the grains. This asymmetric distribution likely results from physical forcing during the naturally occurring sediment transport when the grains are subject to mechanical abrasion^17,46,47^. The locations may also provide protection from grazing pressure by protists^48,49^ as well as viral lysis^50^. The high cell densities in the depressions would also support intra- and interspecies communication and interactions^51–53^.

**Figure 5:**
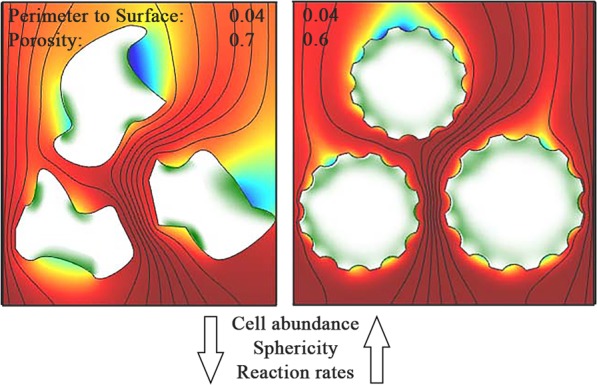
Schematic illustration indicating the microbial colonization and oxygen distribution in the pore space of sandy sediments for grains with different sphericity. Red indicates oxic volumes, blue indicates anoxic volumes and green indicates microbial colonization. The perimeter to surface area ratio is the same between the two cases, but porosity is decreased from 0.7 (left) to 0.6 (right). Based on our results, golf-ball like structures with higher sphericity are better ventilated and thus facilitate microbial colonization. Notice, under *in situ* conditions the porewater flow is highly dynamic and regions of oxia/anoxia might vary.

Although available grain surface area explained cell abundance well, the relationship improves substantially when surface-to-volume ratio was normalized by sediment porosity (Fig. 2b). The porosity at a given surface-to-volume ratio is an indicator for the sphericity of the sand grains^54,55^. A higher sphericity of grains at constant surface-to-volume ratio must theoretically lead to a lower porosity - a relation which was also confirmed by direct measurements (Fig. SI 4a). Even though this relationship seems to contrast with the observation that microbes preferentially colonized cracks and depressions, it is reasonable when considering the ventilation of the pore-space. Spherical grains are likely better exposed to the porewater flow than irregularly shaped grains (Fig. 5), ensuring a better supply of reactive solute compounds and therefore leading to higher growth and cell densities (Fig. 2). Vice versa, irregular grains have surface areas that are potentially less ventilated. Indeed, the FTR experiments showed that enhanced porewater velocities stimulated bulk reaction rates (Fig. 4a) suggesting that some locations in the FTRs were limited by solute transport. This velocity effect was stronger for sediments with high porosity and low grain sphericity (Fig. SI 4), i.e. for sediments with more irregular sand grains which are harder to ventilate. This limitation is likely overcome as porewater velocities and overall ventilation increase. In summary, it seems that an exposed positioning in the porewater flow is of advantage for the microbial community; leading to less dependency on porewater velocities and higher cell densities on spherical grains. Microbes that colonize a sand grain face a dilemma: they must protect themselves from abrasion while at the same time staying sufficiently exposed to porewater flow. Sand that is composed of spherical grains with many small scale local depressions – similar to a dimpled golf ball – would therefore select for the highest cell abundances and rates (Fig. 5).

Normalizing the respiration rates in the 5 FTRs by cell abundance excludes the effects of the heterogeneous cell colonization and singles out the velocity effect. As a result, the cell specific rates for the different cores converge but still show a clear increase with flow velocity (Fig. 4b). When flow velocity is increased, any limitation by electron donors and acceptors will gradually be alleviated, as the supply of electron donors and acceptors increase. At lower flow rates, two limitations seem plausible: either cells become diffusion limited on the micro-scale, as already indicated by the relationship between sphericity and cell abundance, or bulk limitation of electron donor and acceptor supply occurs on the macro-scale along the flow paths in the core.

Bulk oxygen limitation can be ruled out in our experiments due to the experimental design; as we only assessed rates in incubations where the critical Damköhler number was above unity for oxygen. Previous studies were reexamined for regions above the critical Damköhler number and similar slopes of 1.6–2.2 µmol l^−1^ cm^−1^ were found^23,26^. We cannot however rule out bulk limitation of electron donors (i.e. organic carbon), as these would have their own critical Damköhler number depending on the respective concentration. It has been shown previously that the addition of acetate stimulates oxygen respiration in permeable sediments, indicating especially the potential limitation of labile dissolved organic matter^26^. However, the composition of dissolved organic matter is very diverse, and critical Damköhler numbers would have to be established for every individual compound. Further investigations are needed to understand how DOC content and lability affects the reaction rates in sands.

As indicated by the effect of sphericity on cell abundance, the velocity effect may result from diffusion limitation in the microenvironment of the microbial cells, especially those in less ventilated microniches such as cracks and depressions. Higher transport rates result in higher bulk concentrations, i.e. stronger concentration gradients, and hence stimulation of volumetric respiration rates. Considering a single cell reaction rate of R_c_ = 0.04 fmol cell^−1^ h^−1^ and a cell diameter of 0.2 µm, the volumetric cell-specific reaction rate can be estimated to be around 955 mol m^−3^ h^−1^. By assuming a continuous biofilm with a theoretical thickness of 1 µm, diffusion limitation might occur at oxygen concentrations of 20 µmol l^−1^ if the diffusive boundary layer thickness exceeds 100 µm. Considering the low sphericity of sand grains, boundary layer thicknesses of more than 100 µm are likely. A further indication for microscale diffusion limitation is that the velocity effect on cell specific rates is strongest for the sediment fraction with the least grain sphericity (Fig. 4b).

In this study, the difference in sand grain sphericity between the cores was fairly small. This may vary in other regions as the average grain sphericity is directly affected by the amount and age of various mineral components, e.g. chert, igneous rock, or shell fragments. These may result in quite different velocity effects and colonization patterns. Interestingly, the velocity dependent rates in this study never reached their maxima; rather they plateaued, which suggests that the microbial community was still performing far below its optimum. The ability to quickly respond suits the opportunistic lifestyle necessary in sands, where reactive compounds come and go with variations in the flow.

To conclude, within permeable sands the surface area available for microbial colonization has a strong impact on cell abundances and subsequently remineralization rates. Cell densities on individual sand grains are more dependent on the sphericity of the grains, rather than on their size. At smaller scales, microstructures such as cracks and depressions further modulate microbial colonization. In these microstructures the microbial activity is limited either by bulk organic carbon concentrations or by diffusion limitation in less ventilated niches. Empirical relations, as presented throughout the manuscript can express all these relationships. Although the relationships may facilitate the incorporation of permeable sediment processes into biogeochemical models, the further investigation of how organic carbon variability and seasonality influences cell abundance and metabolic rates in marine sandy sediment remains an outstanding task.

## Supplementary information

Supplementary Information.
