# Supplementary material for: The effect of sediment grain properties and porewater flow on microbial abundance and respiration in permeable sediments
Source: Sci Rep. 2020 Feb 27;10:3573. doi: 10.1038/s41598-020-60557-7 (PMC7046789; doi:10.1038/s41598-020-60557-7)
Supplement: Supplementary file 1 — Supplementary Information. [file 41598_2020_60557_MOESM1_ESM.docx]

Supplementary Information: **The effect of sediment grain properties and porewater flow on microbial abundance and respiration in permeable sediments**

## Soeren Ahmerkamp^1,2^, Hannah K Marchant^1^, Chao Peng^1,3^, David Probandt^1^, Sten Littmann^1^, Marcel MM Kuypers^1^ and Moritz Holtappels^1,2,4^


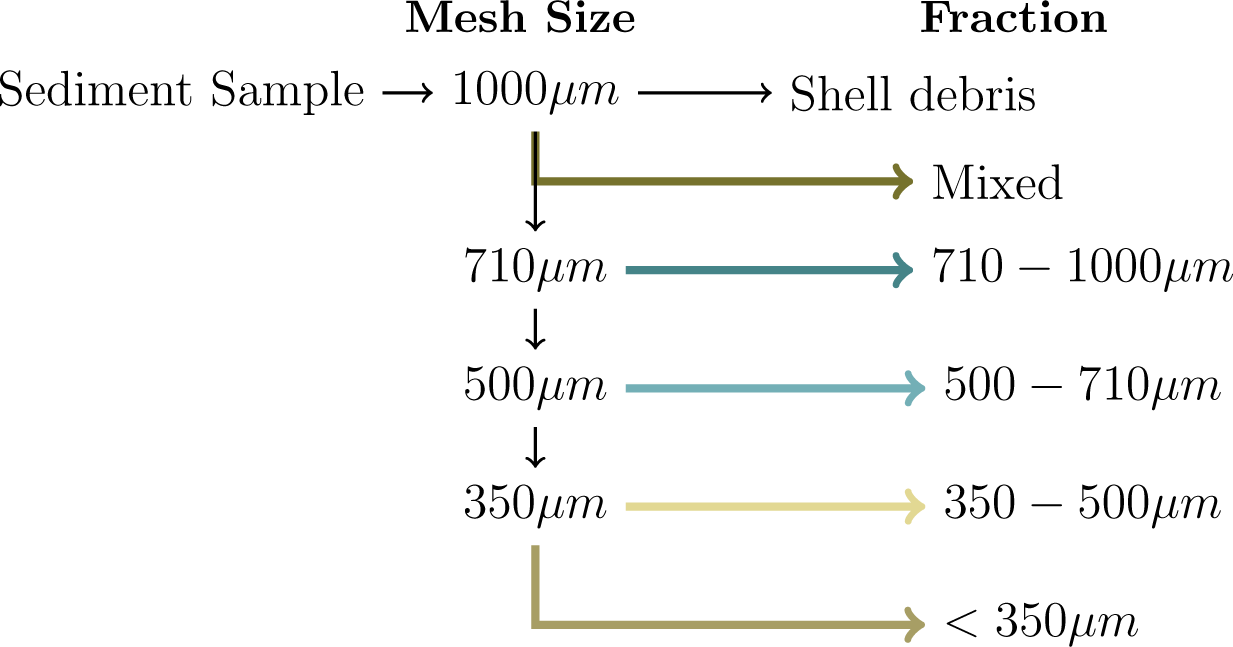


Figure SI 1: Sediment treatment scheme indicating the different sieving steps.


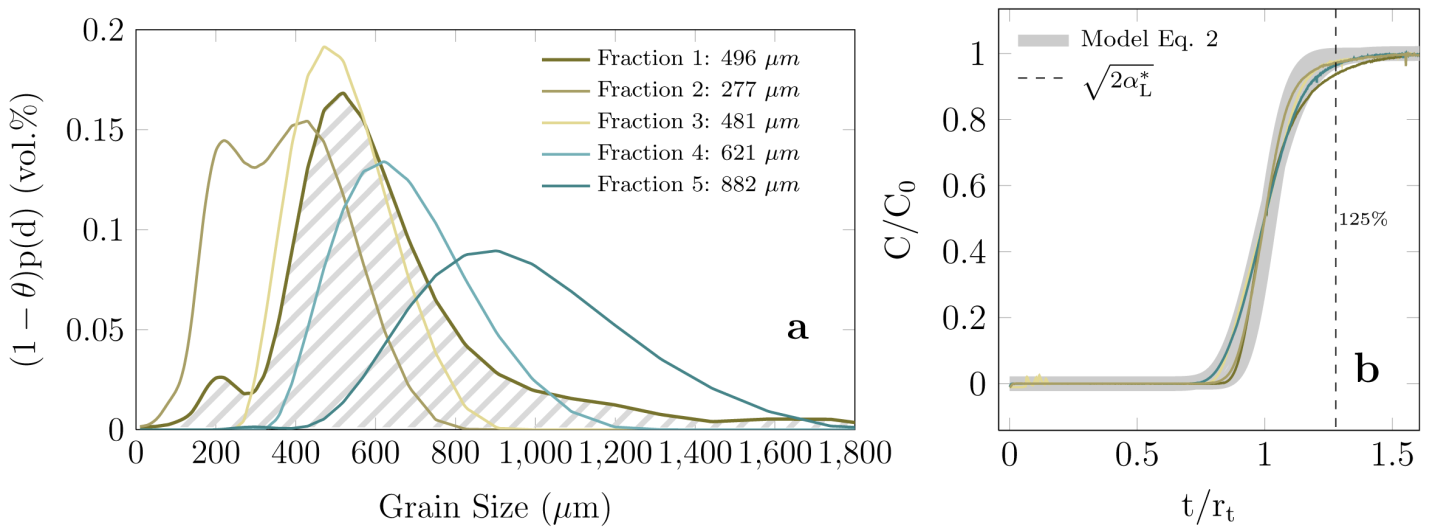


Figure SI 2: Physical properties of the different sediment types. a the sediment distribution of the five different fractions where the shaded distribution indicates the mixed sediment fraction. b breakthrough curves determined by using argon as an inert tracer. The light grey color in the background indicates the breakthrough curves determined from eq. 1 based on the estimated parameters. The dashed line indicates the cutoff used for the Damköhler number – the time to which mixing of different water ages biases rate estimates (see text for more information).


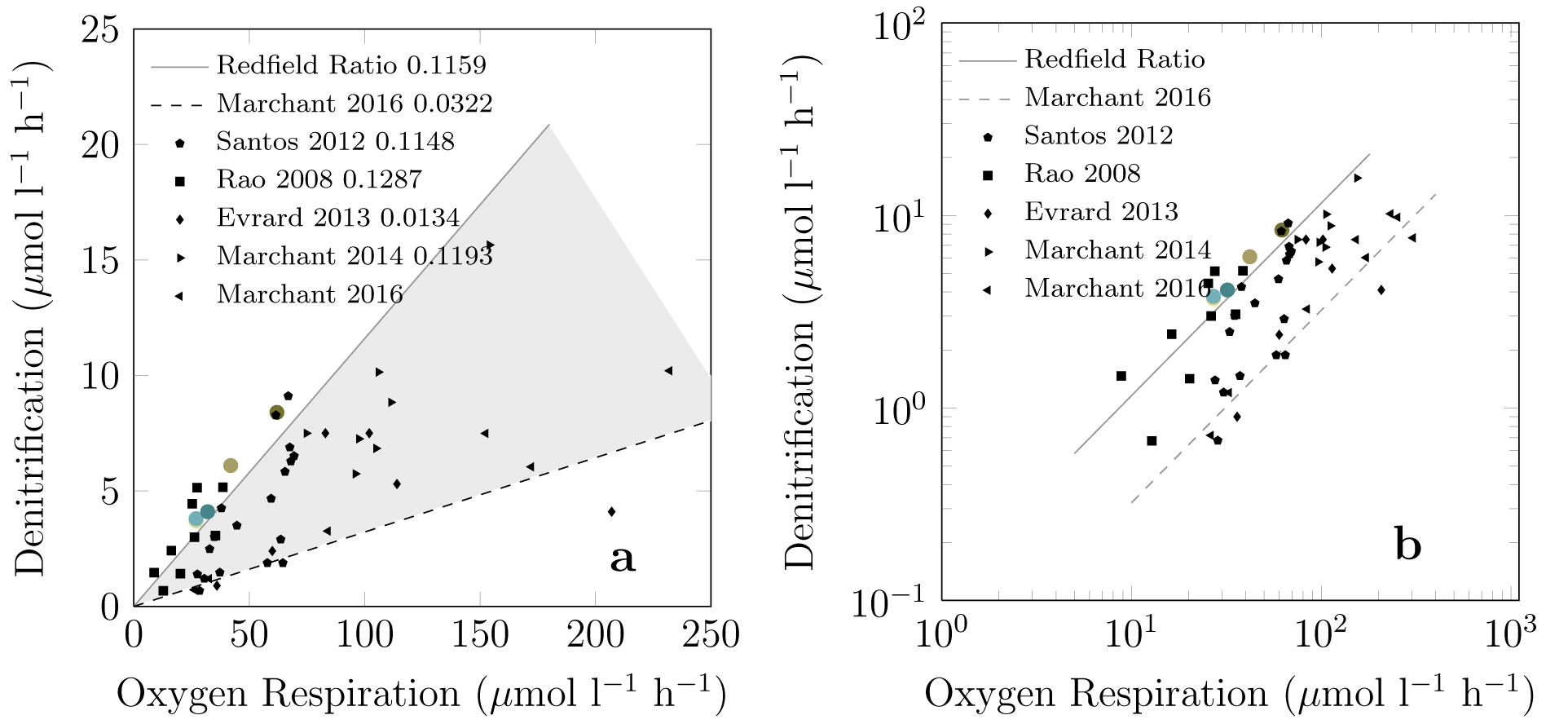


Figure SI 3: Comparison of denitrification rates and oxygen consumption based on the data of various authors.


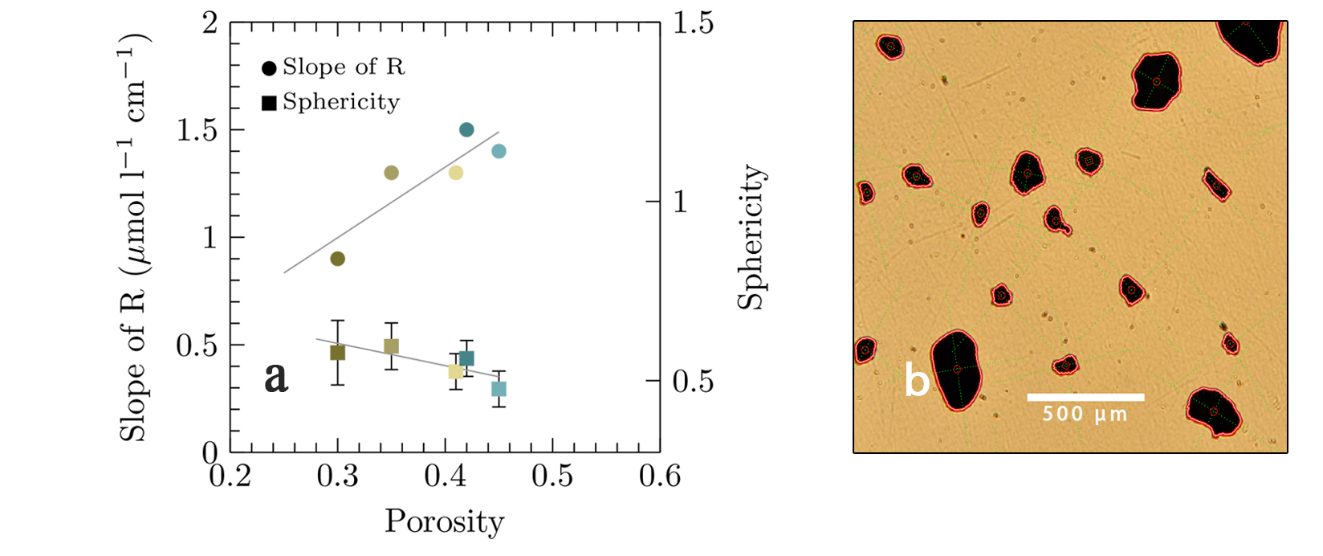


Figure SI 4: a Sphericity (S_P_=-0.6⋅θ+0.02, R^2^=0.61) and slope of R (S_lope,R_=3.2⋅θ+0.02, R^2^=0.75) correlated to the porosity (θ). b Results of the detection algorithm are depicted (see text for further information).

**SI 5 Critical assessment of the respiration measurement**

The respiration rates increased also along increasing porewater velocities in each flow-through reactors (Figure 4 **a**). Two trends become visible when separating the data that fall either below or above the critical Damköhler number (eq. 2, compare Figure 4 open / closed symbols). At low porewater velocities (Dak < 1) the rates were limited by insufficient transport of oxygen into the flow-through reactor. Then parts of the flow-through reactor become anoxic and every increase of pumping speed causes a strong increase of the calculated rates. In this case, calculated rates are underestimated and cannot reflect the actual rates. This effect holds until, the critical Damköhler number is reached (Dak = 1). At Dak > 1 such measurement biases can be theoretically excluded. For the following analysis we only use the data above the critical Damköhler number. At Dak > 1 we still observed a significant positive correlation of rates and porewater velocity although with a much weaker slope.
